# Giant electric-field-induced strain in lead-free piezoelectric materials

**DOI:** 10.1038/srep25346

**Published:** 2016-05-03

**Authors:** Lan Chen, Yurong Yang, X. K. Meng

**Affiliations:** 1 Institute of Materials Engineering, National Laboratory of Solid State Microstructures, Collaborative Innovation Center of Advanced Microstructures, College of Engineering and Applied Sciences, Nanjing University, Jiangsu, China; 2 Department of Physics and Institute for Nanoscience and Engineering, University of Arkansas, Fayetteville, Arkansas 72701, USA

## Abstract

First-principles calculations are performed to investigate the structures, electrical, and magnetic properties of compressive BiFeO_3_ films under electric-field and pressure perpendicular to the films. A reversible electric-field-induced strain up 10% is achieved in the compressive BiFeO_3_ films. The giant strain originates from rhombohedral-tetragonal (R-T) phase transition under electric-filed, and is recoverable from tetragonal-rhombohedral (T-R) phase transition by compressive stress. Additionally, the weak ferromagnetism in BiFeO_3_ films is largely changed in R-T phase transition under electric-filed and T-R phase transition under pressure – reminiscent of magnetoelectric effect and magnetoelastic effect. These results suggest exciting device opportunities arising from the giant filed-induced strain, large magnetoelectric effect and magnetoelastic effect.

Large mechanical response to magnetic-filed or electric-field is required for actuators and sensors. High magnetic-field-induced strain was found in magnetic shape-memory alloy Ni-Mn-Ga based materials, which can be recovered by applying magnetic field and alternatively by mechanical compressive loading^1^,^2^. Piezoelectric materials are another kind of materials provide field-induce strain. Lead-based piezoelectric materials, such as (1–x)[Pb(Mg_1/3_Nb_2/3_)O_3_]–x[PbTiO_3_], (1–x)[Pb(Zn_1/3_Nb_2/3_)O_3_]–x[PbTiO_3_] and morphotropic phase boundary of Pb(Zr_x_Ti_1–x_)O_3_^3^,^4^,^5^ are mostly promising for actuators, sensors and energy-harvesting devices due to its high mechanical response to electric-field^6^. Because of the toxicity of lead, the lead-free piezoelectric materials, such as Ba(Ti_0.8_Zr_0.2_)O_3_−(Ba_0.7_Ca_0.3_)TiO_3_, (Bi_1/2_Na_1/2_)TiO_3_-BaTiO_3_, (K_0.5_Na_0.5_)NbO_3_, (Bi_1/2_Na_1/2_)TiO_3_-BaTiO_3_-(K_0.5_Na_0.5_)NbO_3_^7^,^8^,^9^,^10^,^11^ based piezo-ceramics received more and more interesting. However, these ceramics usually give relatively small achievable strain (the order of 0.5%). The lead-free ferroelectric BiFeO_3_ (BFO) provides large polarization and a strain-driven morphotropic phase boundary (MPB) between tetragonal-like (T-) and rhombohedral-like (R-) phases^12^,^13^. This MPB achieves field-induced strain of 5% by electric-filed induced interconverting between mixed-phase and pure T-phase^14^. However, it is still much smaller than 10% induced by magnetic field in Ni-Mn-Ga^15^. Here, by first-principles calculations, we predict an electric-field induced strain of 10% in epitaxial compressive BFO film due to phase transition from pure R-phase to pure T-phase, this strain can be recovered by mechanical compressive loading in the direction perpendicular to the film.

## Results

### R-phase and T-phase under compressive strain

**Figure 1:**
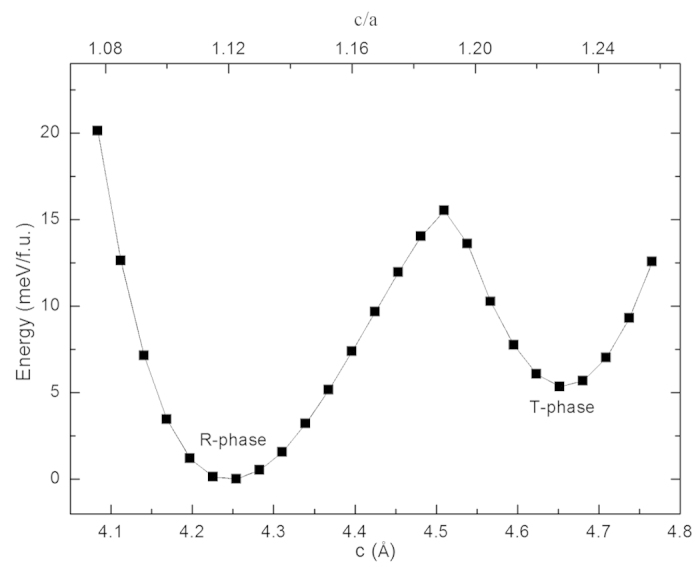
Total energy of BFO film at epitaxial compressive strain 4% as a function of *c* lattice constant. The corresponding axial ratio c/a is also shown. The minimums of the total energy corresponds to the R-phase at c = 4.24 Å and T-phase at c = 4.66 Å.

Under the epitaxial large compressive strain of about 3.5%~5.5%, the R-phase and T-phase have similar total energies and could co-exist^12^,^14^,^16^,^17^. We take the compressive strain of 4% (corresponding to in-plane lattice constant 3.79 Å) as an example to show the properties of compressive films in R- phase and T-phase. As shown in Fig. 1, T-phase has total energy only higher than R-phase by 5.3 meV/f.u. The energy barrier for R-phase transforming to T-phase is 15.5 meV/f.u. and about 10.2 meV/f.u. for T-phase transforming to R-phase. These small activation energy barriers may be easily overcome by external electric filed and stress. T-phase has the minimum energy at out-of-plane lattice constant c = 4.66 Å and R-phase has the minimum energy at out-of-plane lattice constant c = 4.24 Å. These constants corresponds to strain of 10% for T-phase comparing to R-phase.

Table 1 shows the R-phase possesses anti-phase oxygen octahedral antiferrodistortive distortion (AFD) vector of [7, 7, 10]° ([AFD_x_, AFD_y_, AFD_z_] for x-, y- and z-Cartesian components), polarization vector of [0.41, 0.41, 0.76] C/m^2^ ([P_x_, P_y_, P_z_] for x-, y- and z-Cartesian components). We only consider G-type antiferromagnetic (AFM) configuration according the experimental measurement^18^,^19^. R-phase adopts c/a of 1.11, and primary AFM vector along [1–10] direction with magnitude of 4.115 μ_B_, and possesses a secondary ferromagnetic (FM) order having [110] vector with magnitude of 0.019 μ_B_. These AFD, AFM, and FM vector consistent very well with the energy term of 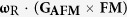, where 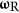 is AFD vector at R-point of the cubic Brillouin zone^20^. T-phase has large axial ratio c/a of 1.23, much larger R-phase. It possesses AFD vector of [0, 4, 4]°, and polarization vector of [0.33, 0.33, 1.28] C/m^2^. These z-components value in AFD and polarization for T-phase is very different than x- and y-comonent, in contrast with those in R-phase where x-, y- and z-Cartesian components are comparable to each other. The primary AFM order of T-phase has [110] vector with a secondary ferromagnetic order having [1–10] vector. Comparing to R-phase, T-phase not only switches AFM vector by 90° and also largely decreases the week ferromagnetism in the magnitude from 0.019 μ_B_ to 0.004 μ_B_. These large differences in AFD, polarization, and magnetic properties between T-phase and R-phase come from the different axial ratio c/a and deformation of oxygen cages.

### R-phase under electric-field

**Figure 2:**
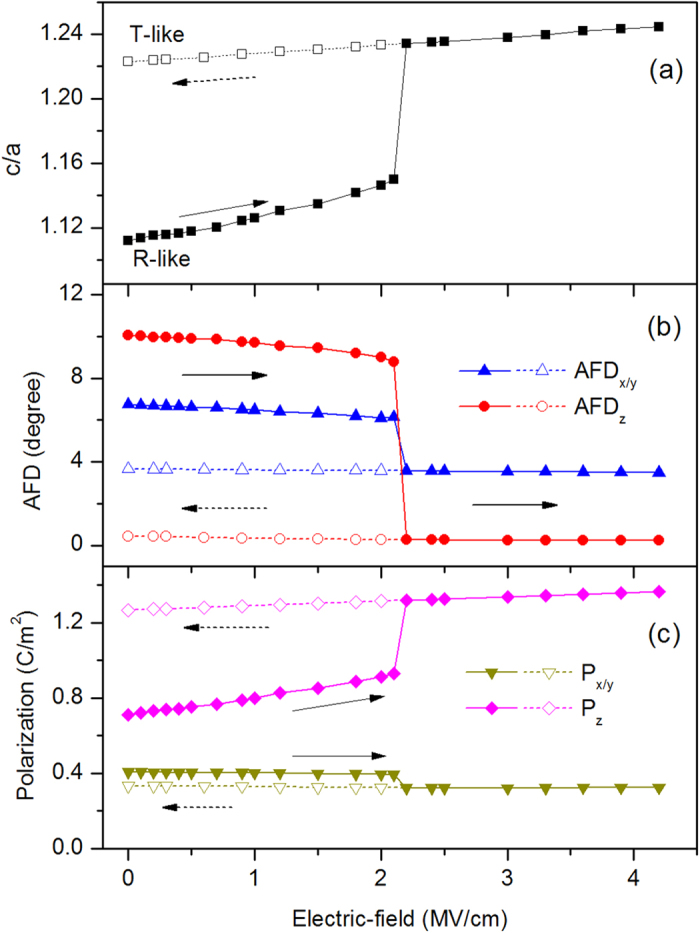
Properties of compressive BFO film under electric-filed along [001] direction. (**a**) Axial ratio of c/a, (**b**) AFD vector and (**c**) polarization as functions of electric-field. The beginning state of BFO under electric filed is R-phase. The filled symbols and open symbols represent increasing and decreasing electric-filed situation, respectively. The x- and y-components of AFD and polarization are equal to each other.

**Figure 3:**
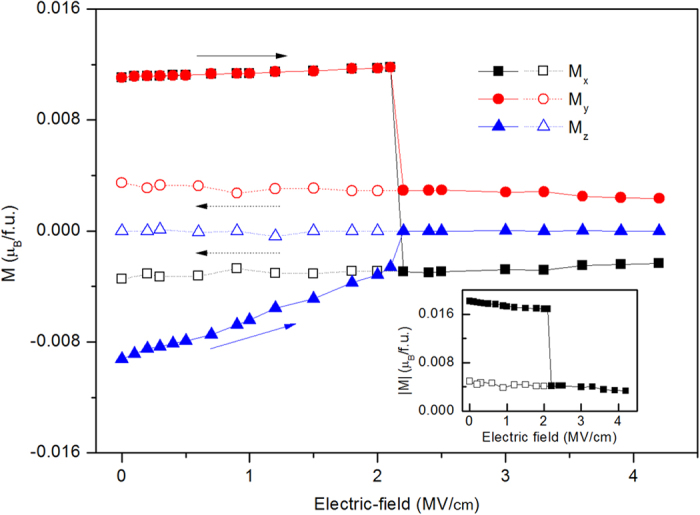
Ferromagnetism of BFO film as a function of electric-field along [001] direction. The beginning state of BFO under electric filed is R-phase. The filled symbols and open symbols represent increasing and decreasing electric-filed situation, respectively.

We now consider situation of R-phase under electric-field and the field-induced-strain. Under external electric field parallel to out-of-plane polarization, one can imagine the out-of-plane polarization would increase and lead to the enhancement of c/a, and finally may lead to phase transition of R-phase to T-phase. Our calculations confirm this imagination of R-T phase transition induced by electric-field. Figures 2 and 3 show the c/a, AFD, polarization, and ferromagnetism under electric field from 0 to 4.2 MV/cm. As the electric filed increases from 0 to 2.1 MV/cm, the BFO film adopts R-like phase and c/a gradually increases from 1.11 to 1.15, ADF vector almost keeps its value unchanging, about [7, 7, 10]°, P_x/y_ remains the value about 0.4 C/m^2^, and P_z_ linearly increases from 0.71 C/m^2^ to 0.93 C/m^2^ due to the response to the electric filed. The AFM vector does not change its direction and magnitude at this electric-field range from 0 to 2.1 MV/cm. x- and y-components of the FM does not change its value of 0.011 μ_B_, while z-component decrease its magnitude from 0.009 μ_B_ to 0.003 μ_B_, as shown Fig. 3. From the linear dependence of magnitude of ferromagnetism on electric-field as shown in inset of Fig. 3, we can get magnetoelectric (ME) coefficient 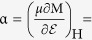 5.1 ps/m. This ME coefficient is very close to that reported in Ref. 21 by a full first-principles scheme, confirming the accuracy of our approximate scheme of studying the responses to finite electric fields. At electric field of 2.2 MV/cm, R-phase of BFO film transforms to T-phase. Note that the R-T transition of BFO under zero strain by external electric field was also found in recent first-principles calculations by constraining electric displacement field^22^, indicating the our approximate scheme of treating electric filed could give similar accuracy with first-principles calculations. This first-order phase transition is characterized by the abruptly jumps of the quantities of c/a, oxygen octahedral tilting, polarization and magnetization. In this phase transition, c/a increases to 1.23 from 1.15, z-component of AFD vector decreases to about 0° from 8.8°, x- and y-components of AFD decreases to 3.6° from 6.1°, z-component of polarization increases to 1.32 C/m^2^ from 0.93 C/m^2^. The primary antiferromagnetic vector is not changed in the magnitude and switched by 90° under this electric field, and the secondary ferromagnetic vector is switched from [uu-v] diection to [-u′u′0] direction, where u = 0.012 μ_B_, v = −0.003 μ_B_, u′ = 0.003 μ_B_. When the electric field further increases to 4.2 MV/cm, the T-phase keeps its most properties unchanging except the slightly increasing of c/a and z-component of polarization. Then, we gradually decrease the electric field from 4.2 to 0 MV/cm, similar to that under increasing electric-filed, the properties of T-like phase are almost not changed under the decreasing electric field. From Figs 2 and 3, the remnant T-phase is stable after releasing electric-field, the tensile strain of 10% of *c* lattice constant comparing to R-phase is reserved, and the magnitude of ferromagnetism is largely decreased.

### T-phase under compressive stress

**Figure 4:**
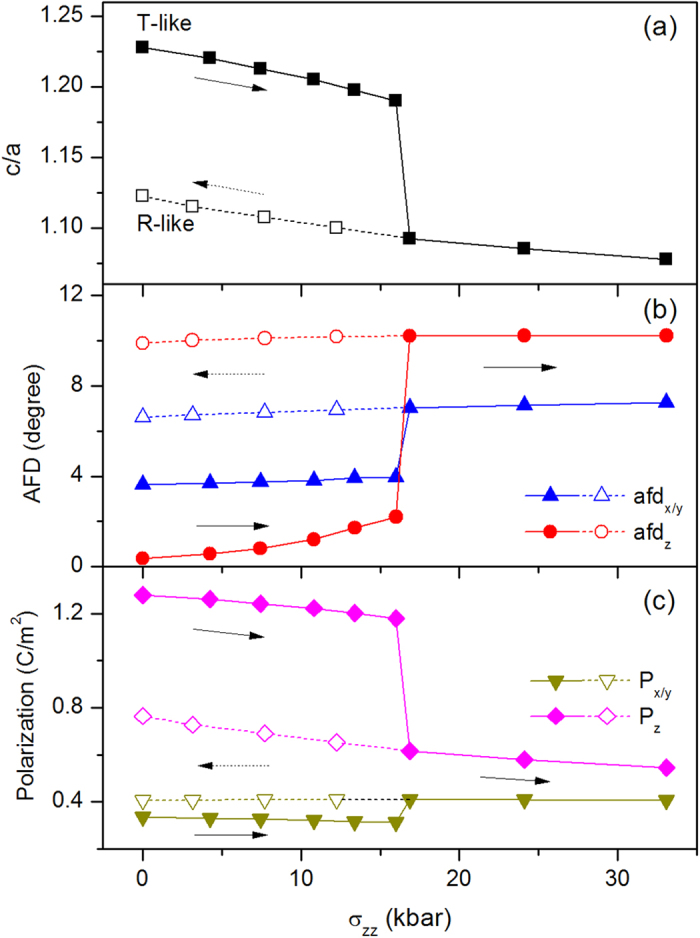
Properties of BFO film under stress σ_zz_. (**a**) Axial ratio of c/a, (**b**) AFD vector and (**c**) polarization as functions of σ_zz_. The beginning state of BFO under stress is T-phase. The filled symbols and open symbols represent increasing and decreasing σ_zz_ situation, respectively. The x- and y-components of AFD and polarization are equal to each other.

**Figure 5:**
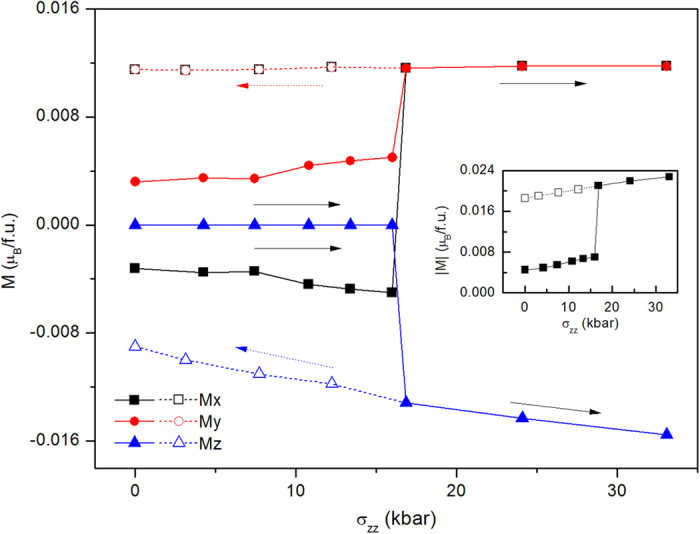
Ferromagnetism of compressive BFO film as a function of stress σ_zz_. The beginning state of BFO under electric filed is T-phase. The filled symbols and open symbols represent increasing and decreasing σ_zz_ situation, respectively.

We now consider how to reverse the tensile BFO film from T-phase to R-phase. If we apply electric field antiparallel to the out-of-plane polarization, the out-of-plane polarization of T-phase would be decreased and finally be switched by 180°, and thus T-phase cannot be transformed into R-phase by this electric-field. One method to recover the BFO film from T-phase to R-phase is compressive stress perpendicular to the film (σ_zz_). Figures 4 and 5 exhibit c/a, AFD, polarization and ferromagnetism as functions of stress (σ_zz_). Under the increasing σ_zz_ from 0 to 16 kbar, c/a of T-phase decreases from 1.23 to 1.19. x/y -components of AFD remain about 4°, and z-component of AFD is very small, lowly increase from 0° to 2°. z-component of polarization slowly decreases from 1.30 C/m^2^ to 1.20 μC/m^2^, while the in-plane component of polarization, P_x_ and P_y_, are almost remain about 0.31 C/m^2^. Similar to the slightly changing of ferromagnetism of T-phase under electric filed, the weak ferromagnetism of T-phase slightly increase under stress 0 to 16 kbar. When stress σ_zz_ increase to 16.8 kbar, T-phase transforms into R-phase (this critical stress can be easily achieved by AFM tip experimentally), which is characterized by c/a abruptly decreases to 1.1 from 1.19, AFD vector jumps to [10°, 10°, 7°] from [4°, 4°, 2°], polarization vector changes to [0.41, 0.41, 0.62] C/m^2^ from [0.31, 0.31, 1.20] C/m^2^, and ferromagnetic vector changes to [0.012, 0.012, −0.013] μ_B_/f.u. from [−0.005, 0.005, 0] μ_B_/f.u. where not only the direction is changed and also the magnitude is enhanced. As the stress further increases from 16.8 kbar to 33 kbar, the BFO film maintains the characteristics of R-like phase, with slowly decreasing of c/a and z-component of polarization, and slowly increasing of the weak ferromagnetism. This magnetic variation with stress corresponds to the so called magnetoelastic effect (inverse magnetostrictive effect), with magnetoelasto coefficient of 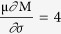 mT/GPa, which is smaller the tranditional ferromagnetic materials^23^. Then we decrease the stress from 33 kbar to 0 kbar, the properties of R-phase remain similar properties with a little changed c/a, z-component of polarization and weak ferromagnetism. Therefore, by compressive stress, T-like phase of BFO film can be transformed into R-like phase, and this R-like phase is remnant when stress is released. The giant electric-field induced strain in BFO film is recovered by T-R phase transition under stress.

## Discussion

Our predicted critical electric filed to switch R-phase to T-phase is 2.2 MV/cm, it may be over-estimated because experimentally it is 0.5 MV/cm to switch mix phase (R and T phases are mixed together) to pure T-phase of BFO experimentally^14^. On the other hand the critical electric filed to switch the polarization of T-phase of BFO is about 2.0 MV/cm^24^,^25^. Therefore, it would be achievable experimentally to switch R-phase to T-phase by external electric filed because its critical electric filed is close or lower that that to switch the polarization of pure T phase. We predicted critical stress to switch T-phase to R phase is 16.8 kbar. Experimentally the pressure on thin film is usually achieved by AFM tip, it is easy to apply a pressure of the order of magnitude of 10 GPa for most AFM tip currently^26^,^27^. Our predicted critical stress of magnitude is 16.8 kbar (1.68 GPa) would be easily achieved in experiments. The BFO film under large compressive epitaxial strain is stable because of strong clamping effect of the substrate. From our calculations, it is difficult to investigate the cycling properties of this transition between R-and T-phases under electric field and pressure. We hope more experimental scientist would be interested and investigate the cycling properties of transition between R and T phases under large strain.

We performed first-principles calculation to investigate the structure, electrical and magnetic properties of T-phase and R-phase BFO films at large epitaxial compressive strain of 4%, and the phase transition between R-phase and R-phase by external electric-filed and stress. We find a reversible giant strain of 10% in this epitaxial compressive BFO film, which is achieved by R-T phase transition under electric-field and T-R phase transition under stress. In addition, concomitant large variations of ferromagnetism between these phase transitions – magnetoelectric effect and magnetoelastic effect – are found. Therefore, these results demonstrate the potential of BFO as a substitute for lead-based materials and suggest exciting device opportunities arising from the giant strain and manipulatable ferromagnetism.

## Methods

Density-functional calculations using the Vienna ab initio simulation package (VASP)^28^,^29^ are performed. To mimic the compressive strained [001] BiFeO_3_ (BFO) films, we use the following lattice vectors: 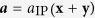, 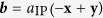,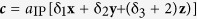, where 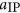 is the in-plane lattice constant of 3.79 Å, which corresponding to the epitaxial compressive strain of 4%. And **x, y** and **z** are unit vectors along pseudocubic [100], [010] and [001] directions, respectively. The supercells used to study the BFO films therefore contain 20 atoms and are periodic along ***a***,***b***,***c*** axes. For the calculation under electric field, the variables of 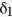,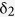 and 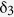, as well as the atomic positions are relaxed to minimize the total energy of 10^−7 ^eV and Hellamn-Feynman forces 0.001 eV/Å on each atoms. For the calculation of relaxation under pressure/stress along [001] direction, the variables of 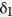 and 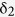 and atomic positions are relaxed, 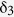 is fixed to mimic certain stress along [001] direction. An energy cutoff of 600 eV and a 6×6×4 Monkhorst-Park k-point mesh wave method were used^3^,^30^. We used the Perdew-Burke-Ernzerhof DFT exchange-correlation functional adapted to solids (PBEsol)^31^ and the projector augmented method to represent the ionic cores^28^. A “Hubbard-U” scheme with U = 4 eV was used for a better treatment of Fe’s 3 d electrons. The calculated lattice constant of ground state by our methods is 3.95 Å, consistent very well with the experiment^32^. Polarization, **P**, is evaluated from the product of the atomic displacements with the Born effective charges. Non-collinear magnetic structure including spin-orbital coupling is considered when calculating the magnetic properties.

An approximate scheme for studying the responses to finite electric fields is used, which starts from the approximate electric enthalpy functional^33^.


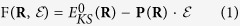


where 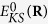 is the zero-field ground-state Kohn-sham energy at coordinates **R**, and **P** is the corresponding electronic polarization. In the presence of an applied electric field 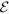, the equilibrium coordinates that minimize the electric enthalpy functional satisfy the force-balance equation


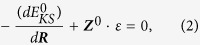


where 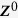 is the zero-field Born effective charge tensor. Such an approximate scheme had been shown to give good accuracy in ferroelectric structures^33^.

We calculated the Born effective charge tensor for both T and R phase by DFPT. Let us label these Born effective charge tensor as Z_T_ and Z_R_ for T phase and R phase, respectively. They are a little different in value. If we compute polarization by using Born charge tensor Z_R_, we could get most feature of the polarization for T and R phases computing by Berry phase. To further increase the accuracy of our calculations, we take a Born tensor Z_RT_ which mix the feature of R-like and T-like phase: Z_RT_(3, 3) = Z_T_(3, 3), and the other components of Z_RT_ are equal to Z_R_. This mixed Born charge tensor Z_RT_ give excellent polarization comparing with Berry phase. For R-like phase, the polarization is [0.38, 0.38, 0.87] C/m^2^ by Born tensor Z_RT_, and [0.39, 0.39, 0.87] C/m^2^ by Berry phase (The polarization vectors show their x-, y-, and z-Cartesian components.). For T-like phase, polarization is [0.31, 0.31, 1.34] C/m^2^ by Born tensor Z_RT_, and [0.35, 0.35, 1.34] C/m^2^ by Berry phase.

## Additional Information

**How to cite this article**: Chen, L. *et al.* Giant electric-field-induced strain in lead-free piezoelectric materials. *Sci. Rep.* **6**, 25346; doi: 10.1038/srep25346 (2016).

## Figures and Tables

**Table 1:** Total energies (E), out-of-plane lattice constant (*c*), anti-phase oxygen octahedral antiferrodistortive distortion (AFD) vectors, polarization (P) vectors, AFM vectors, and FM vectors for R-phase and T-phase.

	E (meV)	*c* (Å)	AFD (°)	P (C/m^2^)	AFM (μ_B_)	FM(μ_B_)
R-phase	0	4.24	[7, 7, 10]	[0.41, 0.41, 0.76]	[2.910, −2.910, 0]	[0.012, 0.012, 0.009]
T-phase	5.3	4.66	[0, 4, 4]	[0.33, 0.33, 1.28]	[2.910, 2.910, 0]	[0.003, −0.003, 0]

These vectors show their x-, y-, and z-Cartesian components.
